# The impact of surgical assistants on postoperative complications in robot-assisted Ivor-Lewis esophagectomy for esophageal carcinoma

**DOI:** 10.3389/fsurg.2024.1492651

**Published:** 2024-11-20

**Authors:** Xipeng Wang, Tong Lu, Wei Guo, Yuqin Cao, Chengqiang Li, Hecheng Li

**Affiliations:** Department of Thoracic Surgery, Ruijin Hospital, Shanghai Jiao Tong University School of Medicine, Shanghai, China

**Keywords:** robot-assisted Ivor-Lewis esophagectomy, surgical assistant, postoperative complication, esophageal carcinoma, thoracic surgery

## Abstract

**Aim:**

This study examines the impact of different surgical assistants on robot-assisted Ivor Lewis esophagectomy. It aims to establish a foundation for refining surgical practices and improving patient outcomes.

**Methods:**

The study included patients aged 18–75 with resectable esophageal squamous cell carcinoma, treated at Ruijin Hospital's Department of Thoracic Surgery (May 2015–November 2023). The robot-assisted Ivor Lewis esophagectomy (RAILE) was executed on a cohort of 97 patients, led by a highly experienced thoracic surgeon and assisted by three additional thoracic surgeons. Postoperative complications, including anastomotic leakage, pulmonary and cardiac events, as well as hemorrhages, were assessed using the Clavien–Dindo classification. The da Vinci Surgical System was used, and statistical analysis was performed using SPSS 20.0, with *P* = 0.05 as the significance threshold.

**Results:**

Of the 97 patients, 50 were in Group A, 23 in Group B, and 24 in Group C. No major differences were found in anastomotic leakage, pneumonia, pneumothorax, severe cardiac complications, chylothorax, and vocal cord paralysis. Assistant C recorded a higher frequency of pleural effusion (45.8%) vs. Assistants A (16.0%) and B (21.7%). The duration of hospital stay was similar across groups, with median durations of 10 days for A, 8 days for B, and 10 days for C.

**Conclusion:**

The study found no significant overall impact of different surgical assistants on postoperative complications in robot-assisted Ivor Lewis esophagectomy. However, pleural effusion rates varied, possibly due to surgical procedure and patient's condition and may be relevant to the assistant's procedure. Future research should involve larger, more varied samples to further validate and refine these findings.

## Introduction

Esophageal carcinoma represents a globally prevalent oncological challenge, occupying the seventh position in incidence and the sixth in mortality among all cancer types (1). This malignancy also features prominently in China's oncological landscape, securing the fourth rank in mortality related to malignant tumors (2). Notably, a significant proportion, nearly 50%, of esophageal cancer cases are identified at an advanced local stage, necessitating surgical intervention as the primary therapeutic approach (3). However, despite the therapeutic advantages of esophagectomy, this procedure is associated with a considerable risk profile, exhibiting complication rates ranging from 26% to 41% and mortality rates between 4% and 10% (4).

The advent of Minimally Invasive Esophagectomy (MIE) marks a significant advancement in reducing postoperative complications and mortality rates, concurrently enhancing the long-term survival metrics, a phenomenon increasingly acknowledged within the academic sphere (5, 6). Concurrently, the realm of thoracic surgery has witnessed a rapid evolution with the integration of robot-assisted methodologies (7). The superior three-dimensional visualization, along with the flexibility and stability afforded by robotic arms, positions robot-assisted surgery as a notable advancement over traditional laparoscopic techniques. Robot-Assisted Ivor-Lewis Esophagectomy (RAILE), an innovation built upon the foundation of the conventional Ivor-Lewis procedure, enables enhanced esophageal exposure, mobilization, lymph node dissection, and facilitates manual anastomoses (8, 9). Within such technically demanding procedures, the role of the surgical assistant, as part of an experienced team (10, 11). Specifically, during robot-assisted anastomosis, the assistant's role in adjusting the stapler angle, adeptly handling the tubular stomach, and coordinating with the primary surgeon to maneuver the robotic arm for precise stapler alignment is of paramount importance, demanding high levels of skill and cooperation.

The influence of surgical assistants in the context of Robot-Assisted Ivor Lewis Esophagectomy remains an area of ambiguity (12). This study endeavors to elucidate this aspect by aggregating data from patients who underwent Robot-Assisted Ivor Lewis Esophagectomy subsequent to the initiation of robot-assisted surgical procedures in the Thoracic Surgery Department of Ruijin Hospital, and aims to analyze the impact of different surgical assistants on postoperative complications.

## Methods

### Data collection

This retrospective study is predicated on an analysis of robotic surgical procedures executed in the Department of Thoracic Surgery at Ruijin Hospital affiliated to Shanghai Jiaotong University, spanning from May 2015 to November 2023. The cohort comprised patients aged between 18 and 75 years, classified with a performance status of 0, 1, or 2 as per the European Clinical Oncology Group guidelines, predominantly presenting with resectable esophageal squamous cell carcinoma (ESCC) [stages cT1–4a, N0-3, M0 or M1 (lymph node metastasis limited to the supraclavicular nodes)], in alignment with the inclusion criteria set forth in the 7th edition of the staging manual by the American Joint Committee on Cancer (AJCC) (13). The exclusion criteria encompassed individuals with neoplasms located in the cervical esophagus or at the gastroesophageal junction, those who were deemed inoperable diseases after evaluation, a history of other malignant conditions, or previous gastric or esophageal surgical interventions.

An exhaustive data analysis was conducted, centering on cases handled with the support of three robotic assistants, identified as Assistant A, Assistant B, and Assistant C. This study encompassed instances where these assistants operated under the supervision of a highly skilled surgeon. Consequently, a total of 97 cases were incorporated into the study, with Assistant A involved in 50 cases, Assistant B in 23 cases, and Assistant C in 24 cases. Postoperative complications, including pulmonary and cardiac events as well as hemorrhages, were methodically classified according to the Clavien-Dindo scheme. Furthermore, specific complications such as anastomotic leak, vocal cord paralysis, and chylothorax were evaluated and classified based on the established consensus definitions by the Esophagectomy Complications Consensus Group (14).

### Surgical techniques

RAILE is performed using a da Vinci Surgical System (Model S; Intuitive Surgical, Inc., Sunnyvale, CA) in our center. The patient is initially supine with a reverse Trendelenburg position for the abdominal phase. The abdominal phase of the surgical approach includes the liver suspension, gastric mobilization with abdominal lymphadenectomy, intracorporal gastric conduit formation, and laparoscopic feeding jejunostomy. The patient is then placed in the left-lateral decubitus position for the thoracic phase with single-lung ventilation. The robot was positioned on the dorsocranial side, with one assistant on the anterior side. CO_2_ insufflation at a pressure of 8–10 mmHg was used for RAILE. The esophagus was mobilized *en bloc* from the thoracic inlet to the gastroesophageal junction, along with all periesophageal lymph nodes. The lymph nodes along the bilateral RLNs were dissected carefully. Both a circular stapled anastomosis and a double-layered, completely hand-sewn intrathoracic anastomosis are used in RAILE.

### Surgical assistants

These assistants, all senior attending physicians affiliated with the Department of Thoracic Surgery at Ruijin Hospital, possess comparable levels of expertise, surgical proficiency, and experience in conducting robot-assisted surgeries for esophageal cancer. They all had participated in a standardized training program for the da Vinci robotic esophagectomy, which included theoretical training on surgical indications, preoperative assessment, surgical steps, and postoperative management; simulation training using robotic simulators for essential skills such as cutting, suturing, and dissection; observation of experienced surgeons to learn intraoperative decision-making and techniques; step-by-step practical involvement under the guidance of mentors. Their training level aligns with that of trained surgeons and they were at the stage of transitioning from simple assistant roles to leading surgeries. Prior to this study, each had performed dozens of robotic-assisted surgeries as the primary assistant and was required to demonstrate proficiency with basic tasks using robotic simulation tools. Besides, they all participated throughout the entire duration of the study.

They play an important role in Robot-Assisted Ivor Lewis Esophagectomy by assisting with instrument setup, patient positioning, tissue retraction, suturing, hemostasis, communication and problem-solving. Their expertise, collaboration, and proactive support contribute to the efficiency, safety, and success of the surgical intervention, and may ultimately benefiting patient care and outcomes.

### Statistical analysis

The data was analyzed using SPSS 20.0 (SPSS Inc., Chicago, IL, USA). Comparisons between continuous variables were conducted using the ANOVA or Kruskal–Wallis test, while comparisons between categorical variables were performed using the Chi-square test or Fisher's exact test. All *P*-values presented in this study are two-tailed, with the threshold for statistical significance established at a *P*-value of 0.05.

## Results

### Patient characteristics

During the course of the study, a total of 97 patients undergoing RAILE were included, with 50 cases (52%) in Group A, 23 cases (24%) in Group B, and 24 cases (24%) in Group C. Demographic and clinical characteristics of the patients were summarized in Table 1 based on the assistant category. No statistically significant differences were observed among the three groups in terms of gender, age, body mass index (BMI), hypertension, diabetes, other comorbidities (including cerebrovascular diseases), smoking history, neoadjuvant therapy, ASA score, tumor location, tumor length, pathological stage and pathological status. These findings provide a comprehensive overview of the demographic and clinical landscape, laying the foundation for subsequent analysis in our study. In the upcoming research, further analyses can explore potential differences among the assistant groups in treatment outcomes, occurrence of complications, and other relevant factors to gain a deeper understanding of the impact of RAILE on different patient populations.

**Table 1:** Baseline demographic and clinical characteristics.

	Assistant A (*n* = 50)	Assistant B (*n* = 23)	Assistant C (*n* = 24)	*P* value
Age (year), median (range)	65 (41–76)	61 (47–76)	67 (51–82)	0.074
Sex [*n* (%)]				0.132
Male	46 (92.0)	22 (95.7)	19 (79.2)	
Female	4 (8.0)	1 (4.3)	5 (21.8)	
BMI (kg/m^2^)	22.7 ± 3.0	23.7 ± 3.0	22.3 ± 2.6	0.248
Hypertension	13 (26.0)	10 (43.5)	9 (37.5)	0.291
Diabetes	8 (16.0)	4 (17.4)	3 (12.5)	0.935
Other comorbidities	6 (12.0)	5 (21.7)	4 (16.7)	0.502
Smoking history	27 (54.0%)	15 (65.2%)	18 (75.0%)	0.214
Neoadjuvant therapy	14 (28.0%)	4 (17.4%)	11 (45.8%)	0.095
ASA score				0.293
Ⅰ	25 (50.0%)	9 (39.1%)	15 (62.5%)	
Ⅱ	25 (50.0%)	14 (60.9%)	9 (37.5%)	
Tumor location [*n* (%)]				0.672
Upper	3 (6.0)	2 (8.7)	0 (0)	
Middle	25 (50.0)	11 (47.8)	14 (58.3)	
Lower	22 (44.0)	10 (43.5)	9 (41.7)	
Tumor length (cm)	2.7 ± 1.2	3.1 ± 1.7	2.5 ± 1.2	0.256
Pathological stage [*n* (%)]				0.594
I	19 (38.0)	4 (17.4)	13 (54.2)	
II	18 (36.0)	8 (34.8)	7 (29.2)	
III	13 (26.0)	9 (39.1)	4 (16.7)	
IVa	0 (0)	2 (8.7)	0 (0)	
Pathological status [*n* (%)]				0.065
pT1N0	19 (38.0)	4 (17.4)	13 (54.2)	
pT1N1	4 (8.0)	0 (0)	0 (0)	
pT1N2	1 (2.0)	0 (0)	0 (0)	
pT1N3	0 (0)	0 (0)	0 (0)	
pT2N0	5 (10.0)	3 (13.0)	3 (12.5)	
pT2N1	5 (10.0)	0 (0)	1 (4.1)	
pT2N2	0 (0)	2 (8.7)	0 (0)	
pT2N3	0 (0)	0 (0)	0 (0)	
pT3N0	9 (18.0)	5 (21.7)	4 (16.7)	
pT3N1	7 (14.0)	6 (26.1)	2 (8.3)	
pT3N2	0 (0)	1 (4.3)	1 (4.2)	
pT3N3	0 (0)	2 (8.7)	0 (0)	

### Postoperative outcomes

The comparative analysis of surgical outcomes among the three assistant groups, encompassing variables such as anastomotic leakage, respiratory system complications, severe cardiac complications, chylothorax, vocal cord paralysis, and length of postoperative hospitalization, is systematically summarized in Table 2. Upon conducting statistical analysis, significant variances were observed in pulmonary complications across the three assistant groups (*P* = 0.002). Notably, Assistant C demonstrated a markedly higher incidence of pulmonary complications (62.5%) in comparison to Assistant A (22.0%) and Assistant B (30.4%). Detailed examination within this category revealed significant disparities in specific complications. A statistically notable difference was identified in the incidence of pleural effusion, with Assistant C recording a significantly higher frequency (45.8%) relative to Assistant A (16.0%) and Assistant B (21.7%) (*P* = 0.005). Conversely, no substantial differences were detected among the groups in the occurrences of pneumonia (*P* = 0.215) and pneumothorax (*P* = 0.633). The incidence of severe cardiac complications was uniformly absent (0%) across all groups (Assistant A, Assistant B, and Assistant C) (*P* = 1.000). Other complications evaluated, including anastomotic leakage, chylothorax, and vocal cord paralysis, did not exhibit statistical significance. Regarding the duration of postoperative hospital stay, no statistically significant variations were found among the three assistant cohorts (*P* = 0.099). The median hospitalization durations were 10 days (range 6–96) for Assistant A, 8 days (range 6–56) for Assistant B, and 10 days (range 6–115) for Assistant C.

**Table 2:** Postoperative outcomes.

	Assistant A (*n* = 50)	Assistant B (*n* = 23)	Assistant C (*n* = 24)	*P* value
Pulmonary complications [*n* (%)]	11 (22.0)	7 (30.4)	15 (62.5)	0.002
Pneumonia	0 (0)	0 (0)	1 (4.2)	0.215
Pleural effusion	8 (16.0)	5 (21.7)	11 (45.8)	0.005
Pneumothorax	3 (6.0)	2 (8.7)	3 (12.5)	0.633
Severe cardiac complications [*n* (%)]	0 (0)	0 (0)	0 (0)	1.000
Anastomotic leakage				0.360
Ⅰ	4 (8.0)	1 (4.3)	1 (16.7)	
Ⅱ	2 (4.0)	4 (17.4)	4 (16.7)	
Chylothorax				1.000
Ⅰ	1 (2)	0 (0)	0 (0)	
Ⅱ	0 (0)	0 (0)	0 (0)	
Vocal cord paralysis [*n* (%)]				0.805
Ⅰ	3 (6.0)	0 (0)	1 (4.2)	
Ⅱ	0 (0)	0 (0)	0 (0)	
Postoperative hospital stay (d), median (range)	10 (6–96)	8 (6–56)	10 (6–115)	0.099

## Discussion

This research elucidates the influence of surgical assistants on postoperative outcomes in patients subjected to robot-assisted Ivor-Lewis esophagectomies. The investigation did not identify any statistically significant differences attributable to the various surgical assistants regarding respiratory complications, including pneumonia and pneumothorax. The sole notable variation was observed in the context of pleural effusion drainage. Furthermore, the study found no significant variations in the incidence of anastomotic leakage, major cardiovascular complications, recurrent laryngeal nerve injury, or the duration of postoperative hospitalization.

An in-depth examination was conducted to elucidate the differential outcomes in pleural effusion drainage attributed to the varying competencies of surgical assistants. Firstly, the proficiency and experience of the assistants may influence the precision and efficiency of the surgical procedure. Assistants with advanced skill levels are likely to execute various surgical steps with greater speed and accuracy, which can help minimize surgical trauma, reduce the postoperative inflammatory response, and potentially decrease the need for pleural effusion drainage. Secondly, variability in preoperative pathological conditions also contributes to the differences observed in postoperative pleural drainage volumes. Factors such as the size and location of preoperative lesions can impact the complexity of the surgery, thereby affecting both the duration of the operation and the prevalence of postoperative complications. However, in our study, despite the observed differences in pleural effusion, the impact of the surgical assistant's experience on this variation appears to be minimal. A plausible explanation for these differences may be attributed to the relatively small sample size, which could limit the statistical power to detect significant associations between the surgical assistant's experience and the occurrence of pleural effusion.

We observed that several retrospective studies have posited that the proficiency level of the surgical assistant does not significantly affect operative duration, perioperative blood loss, or postoperative complications in robot-assisted prostatectomy and robot-assisted partial nephrectomy (11, 15). Concurrently, there exists scholarly work dedicated to assessing the influence of assistant surgeons in robotic-assisted proctectomy (RAP) on perioperative outcomes (16). Within the domain of RAILE, there is a notable paucity of literature that specifically addresses the role and impact of surgical assistants. A particular review highlights that an enhanced technical performance by the bedside assistant in robotic surgeries may contribute to a reduction in operative time, yet it does not necessarily translate into measurable improvements in patient outcomes (17).

This investigation represents a previously unexamined facet of the surgical procedure, carrying substantial implications for clinical practices. The study identified distinct variations in pleural effusion drainage contingent upon the choice of surgical assistant during robot-assisted Ivor Lewis esophagectomy. Such findings underscore the potential impact of the surgical assistant's role on specific aspects of the procedure and the subsequent postoperative results. Nevertheless, this research is subject to several limitations that merit acknowledgment. The limited sample size may potentially diminish the statistical robustness of the findings. Additionally, the disparity in patient distribution across the groups presents a potential bias, which could impinge upon the internal validity of the study. Hence, the outcomes observed should be interpreted with a degree of caution. Moreover, the data being sourced from a single institution might limit the generalizability of these results. Looking ahead, the insights gleaned from this study lay the groundwork for future inquiries and advancements, steering the progression of evidence-based methodologies in the dynamic field of robotic surgical procedures for esophageal cancer. Our research, addressing an aspect hitherto unexplored, establishes a foundational basis for subsequent investigations aimed at refining surgical practices, improving patient outcomes, and contributing to the broader corpus of surgical knowledge.

## Conclusions

We observed no significant distinctions among various surgical assistants on postoperative complications in RAILE. Pleural effusion rates varied, possibly due to surgical procedure and patient's condition and may be relevant to the assistant's procedure. Future research with larger, more diverse samples and consideration of additional confounding factors is crucial for further validation and refinement of our observations.

## Data Availability

The original contributions presented in the study are included in the article/Supplementary Material, further inquiries can be directed to the corresponding author.
